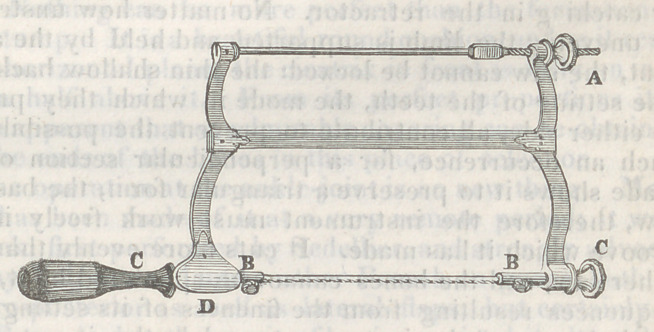# Cases of Amputation

**Published:** 1851-11

**Authors:** Richard G. H. Butcher

**Affiliations:** Examiner on Anatomy and Physiology in the Royal College of Surgeons, Ireland; Surgeon to Mercer’s Hospital, &c. &c.


### SELECTIONS

FROM

### FOREIGN AND HOME MEDICAL JOURNALS.

---

*Cases of Amputation,—1st, below the Knee, subsequently to the Removal of the Foot at the Tibio-tarsal Articulation; and, 2nd, at the Shoulder, during spreading Gangrene consequent on Disease of the Elbow-Joint. The application of a new Saw in both. With Observations.* By Richard G. H. Butcher, F. R. C. S. I., Examiner on Anatomy and Physiology in the Royal College of Surgeons, Ireland; Surgeon to Mercer's Hospital, &c. &c.

William Flynn, aged twenty-five years, a laborer, was admitted into Mercer's Hospital, May 15, 1851. In November, 1849, he had suffered from a severe injury of the foot, which occurred in the following manner:

Having been employed to mind a kiln in which limestone was being burned, he fell asleep at the edge of the crater, when his left leg slipped over the margin on the heated stones; he was quickly awakened to a consciousness of what had occurred, by a sense of severe pain, and, on examination, he found that his foot was very deeply burned, the four inner toes being almost charred, while the little one, together with the tarsus, escaped uninjured. The integuments were also destroyed to the extent of three inches vertically, and two inches transversely on the anterior aspect of the lower part of the middle third of the leg; the thigh suffered slightly on the outer side in its inferior third. As the result of the injury sustained, acute inflammation rapidly followed, particularly in the leg and foot, but of a milder character in the thigh which soon healed, while the leg continued sore for two months before cicatrization was complete, and at the end of three months the toes were cast off from their connexion with the metatarsal bones by ulceration. The wound resulting from this ulcerative process entailed long confinement, and the cicatrix consequent upon its healing was ha.d, unyielding, and sensitive to the touch in the outer half, while that on the inner half, corresponding to where the great toe and the toe next to it were attached, continued painful, inflamed, and discharging a thin sanious fluid. In this condition he continued to move about with the aid of crutches, resting his weight partly upon the heel of the affected limb. In October, 1850, he was admitted into St. Vincent's Hospital, under the care of Dr. Bellingham, who, early in December, performed amputation at the ankle-joint, in the manner recommended by Mr. Syme. Two months after having been dismissed cured from St. Vincent's Hospital, he presented himself for admission at Mercer's Hospital.

He then stated that, from the time of his dismissal, he was never able to lean the weight of his body on the stump, though assisted with crutches; that he freqnently endeavored to habituate the part to gentle pressure, but whenever he attempted to do so, inflammation and increased tenderness of the cicatrix were instantly aroused, which forced him to desist. Not only did the cicatrix become painful, but it ulcerated externally for about an inch. At the same time the cicatrix resulting from the burn in the lower third of the leg again broke out, and assumed the characters of a very irritable form of ulcer, accompanied by excessive pain. From the patient's constantly hopping about on crutches, with the ulcerated leg dependant, it became swollen and œdematous, measuring in circumference above the ankle nearly twice the size of the sound limb. Together with the increase of bulk, the color was of a dark red hue from extreme engorgement of the minutest vessels. Co-existing with this labored circulation, there was a deep heavy pain, particularly elicited on the least pressure over the stump, and if the pressure was made over the inner side of tl e cushion, pain of a very acute character instantly darted up the limb, which as instantly subsided on removing the hand, but could at once be reproduced by the same manœuvre. This test evidently proved that the nerve was involved and productive of it.

The patient came into hospital, and placed himself under my care, with the determination of having his leg removed below the knee, and urged as his reasons the pain he had endured and was suffering, the total uselessness of the limb for progression when he was dismissed from St. Vincent's Hospital with the stump healed, and it being an impediment to his following any mode of business for his support.

In consultation with my colleagues, Mr. Tagert, Dr. Jameson, and Dr. Bevan, they at once acquiesced in my pro osal of amputating immediately below the knee. A few days were allowed to elapse before the operation, in order to facilitate the emptying of the overloaded vessels and the infiltrated tissues of the limb, a result which was readily brought about by position, judicious bandaging, &c. &c.

May 20. The removal of the limb having been decided on this morning, I preferred the method by the double flap operation, and proceeded to execute it in the following manner, preserving about four inches of the leg. The sound leg being secured to the table, standing on the right side of the patient I applied the anterior part of the blade of a long catlin half an inch behind the external edge of the fibula, carrying the instrument downwards, parallel with the bone, for three quarters of an inch, and then sweeping it rapidly in a curve (the convexity downwards) across the front of the leg, until it reached the inner edge of the tibia, when it was directed upwards along its inner margin to the point opposite to where the knife was first laid on, and then, without lifting the blade, the calf was transfixed and the instrument carried quickly downwards, so as to form the posterior flap. The anterior flap, consisting merely of the integuments, was then dissected back a short way, retracted together with the posterior one and retained so by Dr. Bevan, while the amputating knife was swept round and between the bones, as in the circular operation, a little higher than the line of transfixion, thus dividing, posteriorly and in front, the muscular fibres attached to them. A linen retractor was then passed between the bones, and drawn upwards, so as carefully to protect the posterior flap, while the anterior one was dissected up a little way, in order to admit beneath it, to the very line of its osseous connexion, the blade of a fine bow saw, which was placed almost flatly at first on the tibia, and then directed somewhat backwards, so as to cut the bone in a curve (the convexity downwards) to half its depth, the remaining portion, together with the fibula, being severed by working the saw directly backwards.—By this manœuvre it will at once appear that the sharp spine of the tibia was removed in the same section as the rest of the bone. Of the many advantages resulting from the application of this saw I shall speak more fully presently. The anterior tibial, the posterior tibial and peronæal arteries, were then secured; the anterior tibial vessel did not retract, as noticed by some writers, neither was there any difficulty experienced in tying it. The posterior tibial was found and secured more than two-thirds down the flap, and the peronæal ligatured in the usual place, close to the inner edge of the fibula. There not being any other vessels disposed to bleed, the patient was removed to bed, the limb supported on pillows, and the stump exposed to the air to glaze. The operation was performad while the patient was under the influence of chloroform, and its admirable effect in this case cannot be overpraised. In a minute and a half he was placed perfectly under its influence, retained so for a few seconds, and as rapidly roused from insensibility by the fumes of ammonia. The patient awoke to perfect consciousness, entirely ignorant of what had taken place, not having the slightest perception of the least degree of pain during the amputation of the limb.

In four hours after the operation I proceeded to dress the stump; reaction was fully established, and no hemorrhage, venous or arterial, had occurred; the entire surface of the posterior flap was glazed over. The limb could not be touched or the flaps raised, without exciting the greatest torture and violent spasms of the muscles of the thigh. So violent were the patient's struggles, that had we persisted, it is more than probable he would have been seized with convulsions. In this dilemma I at once decided on again putting him under the influence of chloroform. Its anæsthetic effects were as readily induced as in the morning, and then all was calm, perfect immunity from pain having been produced. I quickly proceeded to bring up the posterior flap in apposition with the anterior one, retaining it so by five points of the interrupted suture, and in the intervals applied straps of adhesive plaster; a few folds of fine linen, wet with cold water, were then laid over the face of the stump, which completed the dressings.

The limb was steadied on a pillow, and motion prevented by a broad piece of linen, extending from the knee to the middle of the thigh, and secured by pins to the mattress on either side.

10, P. M. There was slight tendency to starting and spasm in the muscles of the thigh, but he was quite free from pain. He was ordered an anodyne draught.

21st. Slept at intervals during the night, tendency to spasm having ceased almost immediately after the opiate; pulse 88; temperature of the stump a few degrees higher than natural, being moderated by the application of cold. The draught was ordered to be repeated at night.

22d. Slept well; no pain; no startings; no secretion of pus as yet, nor oozing of fluid from the stump; cold to be still continued at intervals, the object being to permit an increase of heat beyond a few degrees; the draught to be repeated at night.

23d. Slept all night, and quite free from pain; the only indication of not being well is the total absence of appetite; pulse quiet, 84.

24th. All tendency to startings in the limb has ceased; there is slight purulent discharge from the inner angle of the wound; I removed one strap of adhesive plaster over this point, and quickly replaced it by another; the sutures are all in position, and there is no trace of irritation about them. Having expressed a wish for food, he was allowed some light farinaceous diet.

25th. Slept the entire night without an opiate; appetite quite restored; pulse quiet; skin cool; the stump presents a most favorable appearance; the outer part of the wound being nearly healed by first intention, I removed two of the stitches corresponding to it; there was not the least blush of inflammation about either, their presence being marked only by a drop of pus following the thread as it was withdrawn; straps of adhesive plaster were applied to support the flaps in lieu of them; the dressings over the remaining portion of the wound in its two inner thirds did not require to be disturbed, there being scarcely any discharge, merely a trace of it along the course of the ligatures. The stump lies still on a pillow, supported as before, merely with its adhesive straps, and remaining sutures: these, together with a few folds of fine linen dampened in cold water, were all the dressings employed.

26th. Slept all night without an opiate, not having experienced pain or uneasiness in the part; all the straps of adhesive plaster, together with the three remaining points of suture were removed to-day; not that the latter were producing any irritation, but that they ceased to further the healing process any more; altogether the stump presents a most favorable aspect; more than the outer third is united by the first intention, and the remainiug portion shows a healthy, granulating surface, not wider than three quarters of an inch in any part between the flaps.

27th. Readjusted the adhesive straps, and placed over them along the track of the wound a strip of lint smeared with zinc ointment.

June 1st. The outer half of the wound is perfectly cicatrized, and along the remaining portion the healthy granulating line between the flaps is not more than half an inch in breadth; new skin creeping in rapidly from both edges on this surface; the ligatures lie still undisturbed.

6th. Progressing most favorably. I gently pulled the ligatures to-day, but each remains undetached.

12th. Wound all healed, though ligatures not cast off.

14th. Ligatures readily came away on traction; stump quite healed.

15th. The stump is now healed throughout, is perfect in every respect, and bears any amount of pressure. When it is placed at right angles with the thigh, the cicatrix is still loose, and a free movement of the integuments permitted over the knee-joint.

I selected the operation by flaps, in this case, in preference to the circular method, not lightly or without thought. For years I have been in the habit of observing the results of each form of operation, and in my own mind, at least, accounting for untoward events, whenever they occurred. After the computation of a large number of cases, results, &c., I am firmly convinced of the superiority of the method by flaps, when effectively and efficiently executed. I am quite aware, when I make so bold an assertion as this, that it is contrary to the predilections of many senior and experienced surgeons, who have been wedded to the circular operation. Should I be considered presumptive for so startling an assertion, I willingly bear the imputation, when I can urge the powerful evidence of Mr. Liston in corroboration of my own views, one whose faithfulness is stamped in imperishable letters on every record which he has left behind. The following remarkable passages occur in the writings of this distinguished surgeon; at page 375 of his Practical Surgery, he says:—"A surgeon must take great pains, and deserves great credit, if he succeeds in making a tolerable stump, more especially when there are two bones, by any other than the mode by flaps; he may cover the bones certainly, but only by integument separated by a painful process from its connexions, and slow, therefore, in contracting new ones." And again, a little before this passage:—"The soft parts and bones not having been well proportioned, the cicatrix, if completed, is tender and liable to ulceration; the ends of the nerves, naturally bulbous when truncated, are exposed and entangled either in the scar or with the end of the bone; and the patient is thereby kept in a constant state of agony. This must be the case very often, so long as the old, round-about, tedious, painful, and imperfect operation continues to be practised. It is true that, in some situations, a good operator can make a very fair and good stump by the circular method, but it is, generally speaking, attended with more suffering, and the results are not by any means so satisfactory."

Further in support of the position I maintain, I will adduce the authority of Professor Fergusson, at page 155 of his Treatise on Practical Surgery. He concludes, after making some observations on both methods, by saying: —"Any one who has had opportunities of contrasting the two modes, must have been struck with the apparent advantages in the execution of the one over the other; the facility of selecting a flap from any convenient side, the comparative ease with which it may be cut, the certainty of preserving a sufficiency of soft parts, the readiness with which the bone can be exposed for the application of the saw, are all, in my opinion, important advantages in favor of the flap operation."

I shall now take a review of the leading features of this case, and show how many of the objections urged against the flap amputation may be guarded against.

As to the position the surgeon should take, when about to perform amputation of the leg, much has been written and said, some contending for one side, while authorities equally as high have proclaimed for the other; the chief grounds upon which the conflicting opinions rest being founded on the anatomical relationship that is known to subsist between the tibia and fibula in their upper parts, the posterior margin of the latter being on a plane considerably behind the broad surface of the tibia; from which it is argued, should the knife be thrust from within, it is liable to be passed between the bones; but the awkwardness that would commit so great a mistake, I conceive, might just as readily consummate the blunder by transfixion from without. On whichever si le of the patient the surgeon stands, it is essential he should remember the oblique surface which the bones, taken together, form posteriorly, and over which the knife is to glide. In the case just detailed, I preferred forming a semilunar flap in front, an inch and a half in length, to the method recommended by Mr. Hey, of drawing the knife straight across the forepart of the leg; and, for these reasons, it meets in approximation more readily the edge of the posterior flap, and lies more evenly in connexion with it; and, above all, when the cicatrix is perfected, and the knee bent at right angles with the thigh, so as to rest in the socket of the wooden leg, the integuments are not strained or tightened. The preservation of the integuments in front then, by the anterior flap, I look upon as a most important feature, bearing on the after condition of the stump; but this of itself will not be sufficient to guard against a dragging of the cicatrix, unless the sharp spine of the tibia be sawn off. I am quite satisfied many of the cases of failure, the ulcerated stumps, &c. &c., which have been ascribed to the weight of the posterior flap, the dragging on the cicatrix by the gastrocnemius muscle (which in reality can have no action at all, when the stump is at right angles with the thigh,) may be more properly ascribed to the neglect of these two points; and the proof I would urge forcibly presents in the fact, that a like sequence not unusually occurs after the circular operation, when the sharp angle of the tibia has been allowed to remain.

In all cases I prefer standing on the right side of the patient; it leaves the left hand free, and in the most favorable position for facilitating the progressive steps of the operation, particularly in holding up the anterior flap, when the spine of the tibia is removed in the manner which I have already adverted to.

The saw which I have used in this operation, and which I most strongly recommend now, for the first time, to the profession, is a modification of the bow-saw used by cabinet makers for cutting out fine work, when curves are to be executed. The construction of the instrument will be readily understood by the description appended to the wood-cut, in which the saw is exhibited on a very reduced scale.

The measurement of the full-sized instrument is as follows: the upright pieces are six inches high, half an inch wide, and two lines thick; the one remote from the handle is received into the transverse bar, and is moveable; the depth of the blade is three lines, with the teeth well set off from each other, and inclined forwards; the length of the blade is six inches, with the sockets included eight inches and a half; the middle bar is half an inch deep, and two lines thick, and the upper bar is rounded, with a screw at one end.*

The instrument, for amputation of the leg and thigh, I have had made of the above proportions, but it is also executed on a smaller scale for minor operations. I have also, at this moment, in the hands of the cutler a saw preserving the same proportions of blade, with a screw to make it tense, directly attached to the remote socket from

---

*A, the nut applied to the screw, which makes tense the blade; B B, the pins that secure the blade in the sockets; C C, the handle and nut, by turning which the blade is rotated to any angle; D, the rest for the index finger.

the handle of the instrument; by this contrivance the upper bar, and a great proportion of the uprights, can be done away with, so as to render it more portable.

The advantages which I conceive this saw possesses over every other, are the following: from the extreme shallowness of the blade, it readily cuts in a curve, if required; and from its slender proportions it can be easily slipped under the flaps and used without bruising them, or catching in the retractor. No matter how unsteadily or unevenly the limb is supported and held by the assistant, the saw cannot be locked: the thin shallow back, the fine setting of the teeth, the mode in which they project to either side, all contribute to prevent the possibility of such an occurrence, for a perpendicular section of the blade shows it to preserve a triangular form, the base below, therefore the instrument must work freely in the groove which it has made. It cuts more evenly than any other saw, and the bones cannot be splintered by it, consequences resulting from the fineness of its setting, and the lightness of the instrument; and lastly, it cuts more rapidly than any other saw, owing to the extreme tension of the blade, produced by acting on the screw in connexion with the upper bar of the instrument; the effect being perfected in a very material manner by the mode in which the blade is riveted in a direct line with the teeth.

This saw will be found extremely useful in every instance where a saw is required, and particularly in amputation of the lower jaw; for, by relaxing the screw above, the sockets in which the blade is lodged are permitted to rotate, so that the teeth may be directed outwards, while, by unscrewing the pin marked B, the blade is readily detached, and, being sharp at the point, is easily passed behind the bone, with its edge applied to it, or at any angle required; the blade is again fastened at B, and when made tense, a few movements of the instrument will readily sever the bone from within outwards.

Again, owing to the facility it affords in cutting curves, it is peculiarly applicable for removing exostosis, cutting out the great trochanter of the femur, &c.

Every surgeon will admit and act upon the grand principle inculcated by modern surgery, to save and protect as much of the body as possible from mutilation; yet in the subject before us, there are two points particularly worthy the attention of every practical surgeon; first, what are the advantages obtained by the amputation at the ankle-joint, and its applicability to all classes of society? and secondly, its terminal results. I have before me at this moment a cast which I procured through the kindness of Dr. Bellingham, taken from the patient, Flynn, immediately on the parts being healed, after the operation at the ankle joint, and before he left St. Vincent's Hospital. Nothing can be more perfect than the formation of the stump. It is a beautiful round cushion, and as it rests on a horizontal plane, the cicatrix in front is fully an inch and a half above it. From its perfect proportions it is quite apparent that an admirable covering can be obtained for the ends of the bones in this place of selection.

The operation at the ankle-joint is no new thing. Mention has been made of it at a very remote period; it was, I think, first performed by Sedellier, and strongly advocated by Velpeau and many other French surgeons, both by antero-posterior as well as lateral flaps; but certainly to Mr. Syme is due the merit of having revived it altogether under a new aspect, by refuting the strong objections urged against it from the extent of the articulating surfaces exposed, and the scantiness of covering for the bones. The former he has lessened by the removal of the malleoli; as first practised by M. Baudens, together with the intervening cartilage; and the latter he has shown can be supplied by an efficient flap from the dense tegument and tissues of the heel. The operation was also strongly opposed on the supposition that the extensor muscles of the ankle, acting through the tendo Achillis, when no longer antagonized, would draw up the heel, and point the cicatrix to the ground. Such a result cannot take place, Mr. Syme says, as the cut extremities of the tendons on the fore-part of the joint speedily acquire new attachments, enabling them to counteract the extensive power. Well, to a certain extent this proposition maintains, perhaps sufficiently so for all practical purposes; yet I have now before me a second cast, taken from the patient Flynn, just before I operated below the knee, an interval of three months having elapsed, and during which time he was hopping about on crutches, with the leg hanging, and the stump in every movement dragged on by the powerful muscles of the calf, not only during his efforts by this mode of progression, but also while he made ineffectual attempts to walk. On contrasting it with that taken on the former occasion, the result contradicts Mr. Syme's assertion that no change takes place in the line of the cicatrix. It is here demonstrated a full half inch lower; but, as I have before noticed, for practical purposes, this need not be taken into consideration, as it is improbable the parts on the anterior aspect of the stump would have yielded any more.

One of the advantages promised by amputation at the ankle-joint instead of the operation near the knee, according to Mr. Syme, is "a more comfortable stump will be afforded." In the case just detailed, we have evidence that the stump was perfectly formed in its most favorable proportions. The patient left the hospital with the cicatrix healed, and, as readily would have been supposed from an inspection of it, complete in every respect; yet what is the disheartening result? Why, that, after weeks of the gentlest trial, the cicatrix breaks out afresh, the limb becomes inordinately swollen, intense and burning pain fixes in the stump, occasioning restless nights and days of torture, and lastly he supplicates for its removal altogether.

The dissection of the stump reveals the causes of all his sufferings, and is extremely interesting. On lifting the indurated integuments, the subjacent layer of granular fat seemed more matted to the fibrous textures, and firmer in its consistence than usual. The insertion of the tendo Achillis was so closely applied to the plantar fascia that it presented the appearance of dividing into three dense fibrous bands, passing from behind forwards to the cicatrix. These bands, on the most convex part of the stump, were separate from each other about half an inch; the spaces between were depressed and filled with fat. On making a section of the cushion from behind forwards, exactly in the mesial line, its depth was fully three-quarters of an inch, its structure fibrous, and eminently springy and elastic; this, together with the integument and fat removed, constituted a covering for the bones of an inch and a quarter thick, and formed of tissues most admirably adapted for the end in view. On examining the cicatrix, the anterior extremities of these fibrous bands, or three divisions of the plantar fascia, were fused into the cut extremities of the flexor tendons, the union between them being short and decided, and hence the improbability that the cicatrix would have descended any more by the action of the extensor muscles. On drawing aside the cut surfaces, it was quite apparent that inflammation, terminating in ulceration, had attacked the cartilage over the articulating surface of the tibia, and also the end of the fibula, from which the external malleolus was removed. The cushion was not adherent to either; it remained firmly attached, however, to the tibia, where the internal malleolus was sawn across. In addition to the increased vascularity of the bones and structures around, there was a delicate adventitious membrane, which was capable of being lifted up, spread out over the eroded cartilage; red vessels permeated and traversed it in all directions, many of them visible to the naked eye, whilst with the assistance of a lens they appeared as a complete vascular network. Coexisting with this condition of the interior, ulceration was also eating its way round the margin of the cartilage. The morbid appearances presented in the parts were brought on, I have no doubt, by the patient's reiterated attempts to move about; and the darting pain occasioned along the thigh, when undue pressure was made at the inner side of the cicatrix, is referrable to the bulbous condition of the nerve, for in this specimen it is very firm, and expanded to the size of a large almond. This pathological condition of the nerve, after amputation at the ankle-joint, is totally at variance with Mr. Syme's prediction when dilating on the advantages of the operation.

In the case just detailed I had no choice of site, the integuments being diseased; if I had, I should have preferred operating at the middle of the leg, or a little below it, the practice comes so strongly recommended from Chas. White, of Manchester, now revived and practised by Prof. Fergusson, of London, and so ably and forcibly urged by Dr. Laurie, of Glasgow; in short, conservative surgery was never so much in the ascendancy as at present, and no man can recognise or estimate the principle more fully than I do. Yet I think it still remains to be proved that the operation at the ankle-joint affords the most comfortable and useful stump to the laboring man, and promises less risk to life. No doubt many cases are on record where the operation was performed, in some instances attended by great success. Lisfranc mentions the case of a man on whom this operation had been performed, who could walk ten or twelve miles a day with great ease, and a few similar cases are recorded by Professor Syme. In most of the published favorable cases, however, we have evidence of a prolonged confinement for the healing of the stump; but after this no reference is made to its usefulness in progression, neither have we full statistics as to the mortality attending it. I have seen the operation performed three times, and in no one case was it attended with success.

Professor Fergusson has written "On the Amputation of the Foot at the Ankle-Joint" in the Medical Times for June of this year, and speaks most favorably of the proceeding; yet he tells us:—"whilst making these observations upon the superiority of this operation, it would not be right in me to lead you to suppose that it is invariably successful, or that it is not sometimes attended by fatal results. Of the eight patients I have operated upon, two died after it; in one of them death followed directly from the proceeding, in a few days, as it would ensue after any other amputation; great irritation and inflammation ensued, and quickly carried the patient off. In the second instance, it would not be fair to put the issue to the operation itself, as the fatal termination did not happen until several weeks after, and it was due to disease of the lungs, which had rapidly supervened after the foot had been removed." This mortality, then, twenty-five per cent., I look upon as very considerable, more particularly when occurring in the hands of this distinguished surgeon. It is greatly to be regretted that Professor Fergusson did not allude, in his lecture, to the present condition of the remaining six of his cases, whether the stump in each case fulfils efficiently the object for which it was intended, and whether any of the patients solicited amputation in preference to being incumbered with a useless limb.

From my own observation, and the facts now detailed, I do not consider this operation at all so applicable to the poor laboring man as to the wealthy sufferer. The latter may at will relieve the stump from pressure, by expensive mechanical contrivance, horse and carriage exercise, &c., &c., whilst the former, no matter what amount of uneasiness he may experience in the part, must endure and struggle on for subsistence, and probably in the end have to submit to another operation.

Michael Scally, aged forty-five years, by trade a carpenter, was admitted into Mercer's Hospital, June 27, 1851. For many years he has suffered from repeated attacks of inflammation of the left elbow-joint, which were variable as to their intensity and duration, but each was followed by considerable impairment of the functions of the part, and usually by marked constitutional disturbance. Twelve days before his admission to the hospital, he was awoke in the night from a sensation of burning pain having fixed in the joint; neither the application of heat or cold, medicated or otherwise, would abate it, and in the morning the affected part was swollen and red, while the limb above and below participated in the discoloration and tension. There was no premonitory sickness of stomach, headache, or fever usherng in this aggravation of the local affection. He applied for relief to a practitioner, but obtained none; and after days of suffering, continuing to grow worse, he was compelled to come to hospital, and seek admission. On presenting himself, he had much the look of a man laboring under far-advanced malignant disease; the expression of the face was indicative of great suffering; it was haggard and sunken, with an icteroid tinge all over it; this color was also remarkably imparted to the sclerotics, at the same time the most striking characteristic of the eyes was their dazzling brilliancy. On being interrogated, he stated that restless nights, with unmitigated suffering, and total disinclination to take food, reduced him to the condition he was then in. On examining the arm, it was swollen round the elbow-joint, four times its natural size as contrasted with the sound limb; the upper third of the fore-arm and the inferior third of the arm were involved in the general swelling; the integuments were of a dark red livid color, with yellowish shading in many places; on pressure the parts were elastic, tense, and shining, while a boggy œdematous track extended along the inner side of the arm nearly to the axilla; the integuments over the deltoid muscle and outer surface of the arm were natural in color and healthy; the constriction of the limb, down even to the fingers, was extreme. Crepitus was quite distinct in the posterior part of the joint, and the olecranon process was moveable.

His pulse was small, rapid, 140 in the minute; and nothing could be more discouraging than the entire aspect of the case. After administering a full stimulant, I proposed immediate amputation of the arm, and this even in very guarded terms, as the only means affording a reasonable hope of saving his life. This proposition would not be acceded to, either by himself or his friends; so then came the question, what mode of treatment next offered the fairest promise of recovery? Amputation being rejected, as a *dernier resort* incision of the limb: yet this held out to me but little inducement to hope for success from its adoption, foı the dan<sub></sub>ers from it presented under two forms, increased depression from the shock, and exhaustion and sinking from loss of blood. Still it was absolutely imperative to free the constricted parts to check the ruin going on and to arrest, if possible, the threatened death of the entire limb. Two incisions were made above the joint, and two below it; so great was the compression exerted on the parts, that the wounds, although made in the long axis of the limb, assumed almost a circular form from the extent to which they gaped. In order to lessen the amount of hemorrhage, and at the same time effectually to relieve the tension, I adopted the following proceeding: having made the first incision, not more than an inch in length, I passed a narrow-bladed knife for an inch and a half or two inches beneath the fascia, with the surface applied to it; then turned the edge forwards, and on withdrawing the instrument, divided the fascia without cutting the integuments. A similar mode was followed in each incision.

Before he was touched with the knife, the patient mentioned forcibly the fact, that whenever he cut himself with his tools, it was almost impossible to stop the bleeding; this he experienced over and over again. In the present instance a large quantity of serum and blood followed the incisions; the hemorrhage was troublesome at first, but was checked after some time by pieces of lint steeped in turpentine passed into the wound, well-formed compresses, and gentle pressure by the hands of assistants. After this the limb was placed in the most advantageous position on pillows, the hand being well raised, and a draught of aromatic spirit of ammonia, camphor mixture, and opium, administered. Throughout the day he was supported with wine, nutritious broth, &c., and the opiate repeated at night.

June 28th. Has had no sleep; raving at intervals through the night; pulse rapid and small; there has been no return of the bleeding; the limb, although lessened in volume on yesterday, after the escape of serum and blood, is to-day tense, engorged as before, and of a livid color; it is extremely sensitive, so that he cannot bear the slightest touch without increase of suffering; and the inflammation, still progressive, has assumed perfectly the gangrenous character, marked by its peculiar discoloration, flaccid bullæ, &c. As one bad symptom, hiccough, had not yet supervened, I again urged the propriety of amputation, as affording, even at so remote a period, a chance of success. My colleagues, Mr. Tagert, Dr. Jameson, and Dr. Bevan, readily acceded to my proposal, and the poor sufferer most urgently wished for it now.

At 11 o'clock, A. M., I proceeded to remove the limb, and adopted the circular method, for reasons to be specified presently. In consultation, it was not considered advisable to place the patient under the influence of chloroform, owing to his enfeebled condition. In a few seconds the limb was removed close to the insertion of the capsular muscles, and during this proceeding not a tea-spoonful of blood was lost from above, owing to the effectual manner in which compression was made upon the subclavian artery by Dr. Jameson, while from the overloaded state of the vessels below the knife, it burst out very freely. Here no bandage could be applied to the limb before the operation, so as to anticipate and lessen this loss, owing to the aggravated suffering which the slightest pressure produced. The axillary artery was secured after it passed the tendon of the subscapular muscle, the posterior circumflex and three minor vessels were also tied; no others at the time required ligatures, or promised any trouble. I considered it the best practice to bring the cut surfaces together at once, from side to side, and retain them in position by a few points of the interrupted suture; the line of union then was vertical, and the lower part of the wound left open for some hours, to permit the escape of any oozing that might take place; the patient was placed in a comfortably heated bed, the stump well supported, and an anodyne draught with ammonia given.

7*

In a few minutes he expressed himself as quite comfortable, and free from pain. In two hours and a half after the operation bleeding began, slowly at first, drop by drop, from the inferior angle of the wound, where it was left open; dossils of lint steeped in turpentine were carefully passed in, gentle pressure applied, and the temperature of the part lowered by cold. After a few minutes it was quite apparent that this would not do; so I at once cut out the stitches, removed the adhesive straps, sponged out a few small coagula, and turned up the face of the stump to the light. No artery in particular was bleeding, but there was a general weeping from the surface; a little blood trickled from the vein, which was readily suppressed by a small piece of lint placed upon the aperture and steadied with the point of the finger for a few seconds, but the welling up from the surface still went on. All the ordinary styptics recommended in such cases were tried, —turpentine, matico, strong infusion of galls, cold air, ice, &c., &c., without the least good effect; the bleeding still continued from almost every point of the divided parts. Under these circumstances, and as the man had lost a great deal of blood, I did not hesitate to apply the hot iron freely over the surface; this even only controlled the bleeding to a certain extent, for the blood still oozed out from the narrow fissures between the muscles; yet by laying fine strips of lint, soaked in turpentine, along their track, and by then applying compresses and gentle pressure, together with a bladder containing ice, over the shoulder and stump, so as to reduce the temperature of the part considerably, the hemorrhage was at length arrested. The amount of blood lost was very great, and as a result the pulse far weaker than in the morning.

9, P. M.—The patient has been liberally supplied with wine and strong broth ever since morning; there has been no return of the bleeding; the application of ice locally, and the wine broth, were continued, the latter even more frequently than in the early part of the day. He remained in much the same state for five hours, when hemorrhage began slowly again. I was instantly by his side, and discovered its source from a particular fissure in the lower part of the stump; he lost about two onnces of blood before it could be arrested; this was affected after a few minutes by compresses steeped in turpentine and retained by gentle and steady pressure with the fingers. His condition now was strikingly characteristic of a man dying from repeated losses of blood; the pulse was scarcely to be felt; the respirations hurried and irregular; intellect clear; voice scarcely audible, yet the one word "air, air," was distinctly pronounced; face remarkably pallid; lips blanched; eyes brilliant; perpetual restlessness and rolling of the head upon the pillow from side to side, and gasping for breath; the hair drenched and the entire body bathed in cold sweat. In this state he continued for about half an hour after the last hemorrhage, when he died.

The heart and large arteries were examined with great care after death, but there was nothing abnormal in their condition; there was no attempt at an internal clot in the axillary artery, yet the blood, which flowed freely in the first instance, on the earliest accession of hemorrhage, and was received in a vessel, coagulated as firmly and as rapidly as under ordinary circumstances. On dissection of the limb, all the soft parts around the elbow-joint were in a perfectly gangrenous condition; the investing capsule of the joint in front was entirely destroyed, and the periosteum was stripped from the lower third of the humerus and the upper third of the radius and ulna. The articulating surfaces of the three bones were entirely denuded of cartilage, and in several places deeply eaten away by ulceration. In many points there was proof of repair being set up at an earlier period, and as if nature struggled hard to effect a cure; for over the articulating surfaces on each bone there were patches of smooth, porcelain-like deposit; the lateral ligaments were destroyed, and the annular ligament of the radius yielded in front. There was a perfect solution of continuity between the olecranon process and the ulna, corresponding to the transverse sulcus lodging the fatty bodies (Haversian glands,) within the joint. It is true the fragments were kept partially in contact by the expansion of the triceps muscle reflected from one to the other, yet the coaptation was not so perfect as to prevent motion between the opposed surfaces, and the crepitus so strikingly elicited during life.

The foregoing case is a well-marked instance of the hemorrhagic diathesis; in its management the most trifling points were weighed to guard against loss of blood.---I should have preferred removing the limb at the shoulderjoint, by a flap operation, and I am convinced it was the most applicable proceeding, when taking into account the diseased condition of the soft parts in the neighborhood of the axilla; yet I readily abandoned it, on estimating the cut surfaces to be greater, consequently the liability to bleeding more than by the circular method.

The case just detailed illustrates well the advantages of the saw represented in the wood cut. The bone had to be divided very high up, and certainly no saw could be passed under the soft parts with a like facility in this situation; the fine narrow blade allowed of this adaptation, and the bone was cut in a perfect curve, a procedure that should be adopted in every case; by it the sharp edges of the bone are removed, which, when left, are a constant inciter to pain and spasm, permitting the soft parts to lie more evenly in contact in the bottom of the wound, and thus facilitating adhesion.

## Figures and Tables

**Figure f1:**